# Editorial: The translation and implementation of robotics and embodied AI in healthcare

**DOI:** 10.3389/frobt.2025.1659302

**Published:** 2025-10-27

**Authors:** Stephanie Tulk Jesso, William George Kennedy, Nele Russwinkel, Levern Currie

**Affiliations:** 1 Department of Systems Science and Industrial Engineering, Thomas J. Watson College of Engineering and Applied Science, Binghamton University, Binghamton, United States; 2 George Mason University Department of Computational and Data Sciences, Fairfax, United States; 3 Group Human-Aware AI, Institut of information Systems, Universität zu Lübeck, Lübeck, Germany; 4 Human IMPAC-T Lab, Industrial and Systems Engineering, Virginia Tech, Blacksburg, United States

**Keywords:** trust in robots and AI, human-centered design, human-machine teaming, translational health research, social robotics and AI, societal impacts, dissemination and implementation science

## Introduction

This paper presents an overview of a Research Topic on the topic of robotics and embodied AI in healthcare. For centuries, scientists and engineers have focused on developing technologies to improve human health. The emphasis is now on finding applications for general-purpose advanced technologies such as robots and AI rather than designing for specific medical needs “from scratch”. Consequently, these solutions may not be aimed at improving human health *per se* but, instead, on improving healthcare efficiency by supporting the human needs of healthcare staff through cognitive support or reduced workload. Still, many questions remain about the true utility of these technologies when implemented within real-world healthcare practice. To respond, this Research Topic attempts to promote an examination of the current level of technological sophistication of robotic and embodied AI tools, current techniques for successful translation outside of the lab, and current evidence on their effectiveness in real healthcare domains. Within the included articles, we find two gaps: one between users’ real-world needs that could be supported with robots and AI and the unrealistic expectations for what such applications could achieve within the complexity of healthcare. Secondly, we see a misalignment between theory and application that leads to a gap between the foundational science and engineering of robots and AI (often labeled the “bench”) and the goal of having useful innovations available for integration into practice. In various ways, the articles within this Research Topic provide insight into both gaps.

## The gap between real-world needs and unrealistic expectations

We suggest that a gap exists between the capabilities of currently available state-of-the-art products and what real users expect and want from robotic and embodied AI applications in healthcare environments. Furthermore, what tasks and roles in the health sector would be a good fit for robots is still an open question. In this topic, Wang et al. surveyed the types of mobile robot systems that have been created to aid nurses, finding 18 robotic products available for home care and healthcare environments. Babalola et al. performed a similar search, finding a general lack of evidence describing collaborative robot platforms that were successfully translated into the world, with many focused on aiding patients rather than directly helping nurses. The works of Wang and Babalola and colleagues are complementary and reveal an important finding: there are more robotic applications sold in healthcare than there are published studies that evaluate whether these tools actually solve real-world problems or demonstrate actual utility. Notably, two robotic platforms that have been implemented in many healthcare systems in the United States, TUG and Moxi, had no peer-reviewed publications produced by the design teams or other groups which presented any evidence on utility or effectiveness. This prompted a follow-up investigation of nurse opinions of these robots expressed on Reddit, finding that users’ perceptions of utility tended to be very negative for Moxi and neutral for TUG (Tulk Jesso et al.). Since the topic of robots in healthcare is extremely popular, it begs the question as to why organizations that produce these robots are not publishing studies to share their evidence. One possible answer is that these platforms fail to demonstrate utility or meet the needs of users within real healthcare environments.

## The gap between “bench” and utility in practice

Traditionally, healthcare innovations were thought to follow a trajectory from “bench to bedside”, following a prescribed pathway from the “bench” of basic science to implementation of new techniques, therapies, and tools at the “bedside”. However, the process is far from straightforward or quick. We suggest robots and AI add complexity and challenges to successful implementation and adoption. Those problems may begin at the onset of a project when designers fail to consider how their products will ensure and demonstrate utility–not in theory, but in real-world practice. A strategy that can help is Human-Centered Design, in which users and other design partners are first consulted about their needs, desires, and the context in which they work at the beginning of the design process. Within the paper by Rafferty et al., which received a best paper award in Frontiers in Robotics and AI in 2024, the researchers described human-centered design research with nurses to determine how AI and robots could help prevent bathroom falls. They found that the toileting task, while imagined as a nuisance for nurses, was perceived as a valuable nurse-patient touchpoint which nurses sought to ameliorate rather than turn over to a robotic helper. They also note the need to consider real user concerns about the potential unintended consequences of these technologies. The current state of robotics is still extremely simplistic, and autonomous robots cannot perform well in complex, dynamic human environments or during social interactions. For real-world utility, human partners need robot and AI system behaviors to be easily understood, and likewise these systems need to be able to infer human partners’ intentions and understand common tasks while avoiding becoming an obstacle. For this, we will need more focus on human-aware AI to sense and understand the situation and then provide appropriate and acceptable roles for the robots. In this vein, Zhang et al. reviewed what kind of sensor systems are available and successfully used to enable reliable awareness of the situation, humans’ intentions, changes, and which possibilities need further evaluation. Zamprogno et al. went a step further by developing a human-sensing robotic system and studied what skills and capabilities (high- and low-level cognitive functions) would be necessary for the robot to anticipate intentions or to offer support in a task without a well-defined result.

## Conclusion

The study and application of technology to improve human health will certainly continue. This Research Topic describes a current focus on applying robotics and embodied AI to healthcare. Many questions remain about the advances’ true utility and this Research Topic attempts to provide evidence to help bridge the two gaps we discovered. The articles describe current practices and necessary research. Our next steps are to articulate the process by which innovations are maximally set up for successful translation and implementation and the various pitfalls observed that lead efforts away from the necessary steps towards success.

